# The prevalence and years lived with disability of asthma in children under 5 years old in Sichuan Southwest China, 1990–2019: A cross-sectional study

**DOI:** 10.3389/fped.2022.899519

**Published:** 2022-08-15

**Authors:** Yu Luo, Li Bao, Mu Wang, Hui Guo

**Affiliations:** ^1^Department of Obstetrics Nursing, West China Second University Hospital, West China School of Nursing, Sichuan University, Chengdu, China; ^2^Key Laboratory of Birth Defects and Related Diseases of Women and Children, Ministry of Education, Sichuan University, Chengdu, China; ^3^Outpatient Blood Collection Nursing Unit, West China Hospital, Sichuan University, Chengdu, China; ^4^Outpatient Department, Mianyang Central Hospital, School of Medicine, University of Electronic Science and Technology of China, Mianyang, China; ^5^Department of Pediatrics, West China Second University Hospital, Sichuan University, Chengdu, China

**Keywords:** children, asthma, YLDs, prevalence, death rate

## Abstract

**Objectives:**

To analyze the prevalence and Years Lived with Disability of Asthma in children under 5 years old and the time trends in Sichuan Southwest China from 1990–2019.

**Methods:**

Based on the results of the Global Burden of Disease Study 2019 (GBD2019), we present the time trend, sex specific prevalence, Years Lived with Disability (YLDs) and deaths due to asthma under 5 years old in Sichuan and compared them with national indicators in the same period.

**Results:**

The number of children under 5 years old with asthma decreased overall from 1990 to 2007 and increased overall from 2008 to 2019. The time trend of prevalence rate was similar. In 2019, over 111 thousands children under 5 years old suffered from asthma, the prevalence rate was 2,584 per 100 thousand, YLDs was 4.5 thousands, and YLDs rate was 105 per 100 thousand in Sichuan. Compared with 1990, the number of patients decreased by 48.6%, the prevalence increased by 9.4%, the YLDs decreased by 48.9%, and the YLDs rate increased by 9.7% in 2019. The increase in the prevalence and YLDs rate of asthma children in Sichuan was higher than the national overall, but the number of asthma deaths and deaths rate in Sichuan and the whole nation all decreased.

**Conclusions:**

The results in Sichuan Southwest China show the prevalence and YLDs of asthma children under 5 years old increased over the past 30 years, and were higher than the overall increase in China. Male children are the key population of increasing asthma disease burden and deserves more attention. More targeted prevention and control measures are still needed to reduce the incidence of asthma.

## Introduction

Asthma is the most common chronic disease in children all over the world, affecting about 14% of children globally with rising prevalence (1). Children's asthma has the characteristics of long course, repeated attack and long-term treatment. It is caused by the expansion and narrowing of the air delivery pipe in the lung, which will cause sporadic dyspnea and even death. More than 80% of asthma related deaths occur in low- and middle-income countries (2). Through correct treatment and management, asthma can be well-prevented and controlled.

Thus far, most of the reports on the prevalence of asthma are over 14 years old, including the prevalence, control rate and cognitive level (3, 4), and relatively few reports on the prevalence of children. The various risk factors studied on the prevalence of children may also become irrelevant depending on the regional differences (5), making it difficult to summarize a general conclusion. The prevalence of childhood asthma in China has increased significantly since the 1990's, and preschool is the age group with the highest prevalence of childhood asthma (6, 7). Some studies have shown that increased airway smooth muscle mass in severely wheezing children under 4 years age can gradually diminish the effectiveness of bronchodilators. And by school age (>6 years old), the thickening of the reticular basement membrane in children with severe asthma is similar to that of adults. These studies suggest that interventions to alter the natural history of asthma would have the greatest likelihood of success if applied in the 1st years of life (8). Hence, early interventions can avoid the delayed development of adult asthma due to untimely or improper treatment, lung function damage, or even complete loss of physical activity (9).

Given that geographical, environmental and social factors can affect the prevalence of asthma, there is a need to summarize the characteristics of asthma of child under 5 years old in Sichuan. Sichuan Province is located in southwest China. The province's landforms vary greatly from east to west, and the terrain is complex and diverse. The western part is plateau and mountainous; the eastern part is basin and hills. The basin is the most densely populated part of Sichuan, with a warm and humid climate, and is also the political, economic and cultural center of the entire Southwest China (10). The unique geographical features makes our study very representative for Southwest China. In this paper, we analyzed the time trend, sex specific prevalence, Years Lived with Disability (YLDs) and deaths due to asthma under 5 years old from 1990 to 2019 in Sichuan Southwest China, and compared them with national indicators in the same period. We hope that by analyzing the regional characteristics of children asthma prevalence and YLDs in Sichuan, we can speculate possible reasons and thus provide a reference for a more targeted plan for children in this region.

## Materials and methods

In this article, we used the Global Burden of Disease Study (GBD) database and collected all research acceptable data on children under 5 years old with asthma in Sichuan province and China between 1990 and 2019. For China's data sources (mainly before 2019), the death data mainly comes from China Disease Surveillance Points and Death Registration, and the non-fatal data mainly comes from the national survey, research and investigation reports published in the scientific literature, such as China World Health Survey. The data included in the analysis are strictly controlled to ensure the reliability of data quality (11).

The methods of the GBD have been reported somewhere else, and the research results have been widely used (12–14). All values are presented with 95% uncertainty intervals (UI) to estimate the distribution based on all data and input parameters including all sources. Non-fatal health outcomes including prevalence estimates and uncertainty distributions were estimated by DisMod-MR 2.1, a developed Bayesian meta regression algorithm calculation engine for GBD research (15). Disability weights were generated using collected data based on household and open internet surveys in 10 countries (Bangladesh, Indonesia, Peru, USA, Tanzania, Hungary, Italy, the Netherlands, and Sweden), all the participants were aged 18 years or older. It is described elsewhere (16). YLDs was calculated by multiplying the prevalence of each sequela by the disability weights, which is between 0 and 1 to quantify the relative severity of the sequela.

We use prevalence rate to reflect the overall severity of the disease in the region, YLDs to reflect the impact on the population quality of life. For children aged 0–14 years, the risk of death due to asthma can be reasonably avoided by health care (17), so we use the mortality rate to reflect the local medical penetration level. The definition for asthma was a reported diagnosis by a physician, with wheezing in the past 12 months.

## Results

The present paper reports prevalence, YLDs and deaths due to asthma under 5 years old between 1990 and 2019 in Sichuan. In 2019, over 111 thousands children under 5 years old suffered from asthma, the prevalence rate was 2,584 per 100 thousand, YLDs was 4.5 thousands, and YLDs rate was 105 per 100 thousand in Sichuan. Compared with 1990, the number of patients decreased by 48.6%, the prevalence increased by 9.4%, the YLDs decreased by 48.9%, and the YLDs rate increased by 9.7% in 2019 (Table 1).

**Table 1:** Asthma prevalence and YLDs under 5 years old, 1990 to 2019 with percentage change.

**Indicators**	**Gender**	**China**			**Sichuan**		
		**1990**	**2019**	**%Change**	**1990**	**2019**	**%Change**
No. of cases (95%UI, thousands)	Male	1606.1(904.2–2620.2)	1240.7(684–2039.4)	−22.8	128.2(73.8–205.7)	70.1(39.6–112.8)	−45.3
	Female	1179.8(650.8–1943.5)	808.8(430.9–1340.4)	−31.4	89.3(46.6–150.9)	41.8(21.8–71)	−53.2
	Both	2785.8(1544–4570.1)	2049.5(1112.8–3400.7)	−26.4	217.5(121.2–356.3)	111.9(61.6–184.1)	−48.6
Prevalence rate (95%UI, per 10^5^)	Male	2635.8(1484–4300.3)	2828(1559–4648.6)	7.3	2639.8(1519.2–4235)	3023.5(1708.7–4864.2)	14.5
	Female	2166.3(1194.9–3568.6)	2150(1145.4–3563.2)	−0.8	2053.2(1071.4–3467.9)	2077.7(1086–3531.7)	1.2
	Both	2414.2(1338–3960.4)	2515(1365.5–4173.1)	4.2	2362.7(1316.2–3869.9)	2584.1(1422.8–4251.8)	9.4
YLDs (95%UI, thousands)	Male	65(32–119)	50.4(24.7–94)	−22.5	5.2(2.4–9.5)	2.8(1.4–5.3)	−46.2
	Female	47.8(23.5–87.1)	32.9(16–60.8)	−31.2	3.6(1.7–6.7)	1.7(0.8–3.3)	−52.8
	Both	112.7(55.5–205.9)	83.3(41.1–154.6)	−26.1	8.8(4.1–16.2)	4.5(2.2–8.6)	−48.9
YLDs rate (95%UI, per 10^5^)	Male	106.6(52.5–195.3)	114.8(56.4–214.2)	7.7	107(50.4–196.2)	122.6(60.1–227.5)	14.6
	Female	87.7(43.2–159.9)	87.4(42.4–161.6)	−0.3	83(38.9–153.5)	84.8(39.6–164.1)	2.2
	Both	97.7(48.1–178.4)	102.2(50.5–189.8)	4.6	95.7(45–176.1)	105(50.5–199.5)	9.7

**Figure 1:**
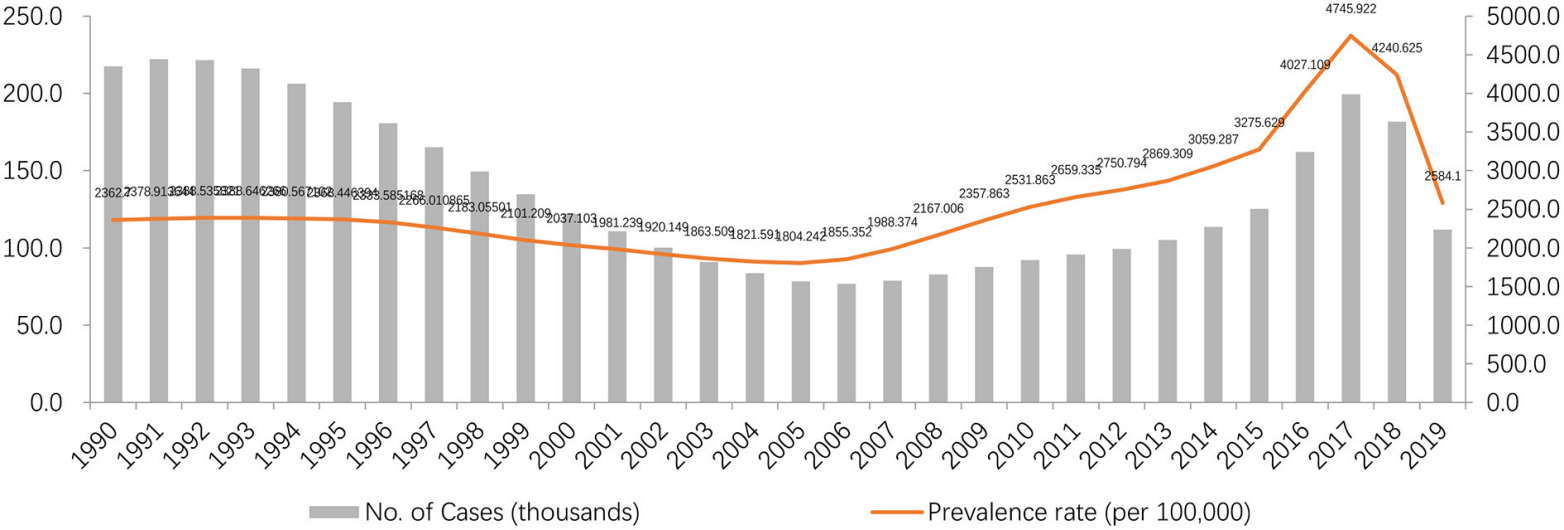
Time trends of prevalence and the rates attributed to asthma under 5 years old from 1990 to 2019 in Sichuan.

Figure 1 shows the trend of cases and prevalence rate during 1990 to 2019 in Sichuan. From 1990 to 2007, the number of asthma patients in children under 5 years old decreased from 218 thousand to 79 thousand, and from 2008 to 2019, the number of asthma patients increased from 83 thousand to 112 thousand. While the prevalence rate was dropped from 2,363 per 100 thousand in 1990 to 1,855 per 100 thousand in 2006, and increased from 1,988 per 100 thousand in 2007 to 2,584 per 100 thousand in 2019.

Tables 1, 2 show the overall prevalence, YLDs and deaths due to asthma under 5 years old and the change by sex in China and Sichuan. In the past 30 years, due to the decline of the birth population, although the number of patients decreased, the prevalence rate increased. The prevalence of asthma in Sichuan province has increased by 14.5% for boys and increased by 1.2% for girls; in the same period, the prevalence of asthma in China has increased by 7.3% for boys and decreased by 0.8% for girls. YLDs rate of boys increased by 14.6% and that of girls increased by 2.2% in Sichuan, while it increased by 7.7% in male and decreased by 0.3% in female in China. The number of asthma deaths and deaths rate among children under 5 years old in Sichuan and the whole nation all decreased.

**Table 2:** Asthma deaths and deaths rate under 5 years old, 1990 to 2019 with percentage change.

**Indicators**	**Gender**	**China**			**Sichuan**		
		**1990**	**2019**	**%Change**	**1990**	**2019**	**%Change**
No. of deaths (95%UI)	Male	416(243–582)	13(8–21)	−96.9	45(13–93)	1(0–1)	−97.8
	Female	409(149–612)	9(6–13)	−97.8	51(10–120)	0(0–1)	−100.0
	Both	825(449–1143)	22(15–31)	−97.3	96(31–181)	1(1–2)	−99.0
Deaths rate (95%UI, per 10^5^)	Male	0.7(0.4–1)	0(0-0)	-	0.9(0.3–1.9)	0(0–0.1)	-
	Female	0.8(0.3–1.1)	0(0-0)	-	1.2(0.2–2.8)	0(0–0)	-
	Both	0.7(0.4–1)	0(0-0)	-	1(0.3–2)	0(0–0)	-

## Discussion

Asthma are important contributors to the burden of non-communicable diseases (NCDs). Although most of the burden can be prevented or treated through affordable interventions, these diseases receive less attention than other NCDs, especially in underdeveloped areas (18) like Sichuan, a province in Southwest China with a developing economy.

According to the GBD data of 2019 in Sichuan Provincein Western China, in recent 30 years, the prevalence of asthma and YLDs rate in the whole population of Sichuan Province has increased slightly, including the rise of men and the decline of women, and the prevalence of asthma and YLDs in children under 5 years of age increased more than the national average. Possible reasons for higher prevalence than average may include high smoking rates, the humid climate of the basin, the environmental pollution that accompanies irrational industrial development, which are all risk factors for asthma in children under 5 years old (19), and the increased attention paid by parents to their children's health as a result of the improvement of education, which may also lead to higher attendance rate. Meanwhile, decreased death rate, combining with the fact that asthma is more prevalent and severe in young boys (20), resulted in significantly higher YLDs rate in boys compared to girls. The non-fatal disease burden caused by asthma in male was always higher than that in female, which is similar to the situation of children reported in many cities in China (21). Therefore, the asthma situation of male children under 5 years old in Sichuan Province deserves more attention. According to the time trend, both the prevalence and the number of YLDs under 5 years old with asthma have declined, which is mainly related to the decline of birth rate in China. While, the prevalence decreased from 1990 to 2007, and increased rapidly from 2007 to 2019 (Figure 1). But the situation may change after the opening of the two child policy in 2016 (22).

Although asthma, if well-controlled, rarely has serious consequences. If the treatment is not standardized and timely, it may still cause sudden death, growth retardation, thoracic deformity, respiratory failure and other serious or long-term harm. The change of death level can reflect the change of medical penetration level in a place. From Table 2, we can see that the number of children 5 years old and below in Sichuan and the whole nation who died of asthma has decreased significantly. From this dimension, we can see that in the past 30 years, the medical penetration level of all parts of the country has been improving.

5-year-old children do not have the ability of independent behavior. The prevention and treatment of asthma in preschool children depends on the guardian's active help. With the aggravation of prevalence and disease burden, it is very important to improve the children's guardians' asthma knowledge level for prevention and treatment. In addition to the promotion of children's passive gymnastics, outdoor activities, fresh air and other knowledge among child guardians, breastfeeding and smoking control should also be advocated by government. Also, government policies to provide a healthy public environment are equally important. More people will benefit from the raising government awareness in developing regions.

In this study, GBD 2019 data and comprehensive modeling method are used for effective comparative analysis. However, there are some limitations in GBD study, such as 95% uncertainly interval of the number is relatively high, data sources used to estimate asthma are not comprehensive enough, some locally sources of asthma data have been ignored. As for China, it does not include survey data published in some Chinese journals, which may underestimate the disease burden of asthma. Because there are few deaths caused by asthma, it is necessary to establish a disease incidence and disease monitoring system in the country.

## Conclusion

Overall, the results in Sichuan Southwest China show the prevalence and YLDs of asthma children under 5 years old increased over the past 30 years, and were higher than the increase in overall China. These may be related to smoking, environmental pollution, and climatic characteristics of the Sichuan region. Male children are the key population of increasing asthma disease burden and deserves more attention. Although the deaths rate of asthma is decreasing, more targeted prevention and control measures by government are still needed to reduce the incidence of asthma.

## Data availability statement

The original contributions presented in the study are included in the article/supplementary material, further inquiries can be directed to the corresponding author/s.

## Ethics statement

Ethical review and approval was not required for the study on human participants in accordance with the local legislation and institutional requirements. Written informed consent to participate in this study was provided by the participants' legal guardian/next of kin.

## Author contributions

YL had full access to all of the data in the study and takes responsibility for the integrity of the data and the accuracy of the data analysis. YL and MW conceived the study design and the analytical plan and prepared the first draft. HG finished the draft based on comments from other authors. All authors contributed to the article and approved the submitted version.

## Funding

HG received grants from the Science and Technology Bureau of Sichuan province (No. 18ZDYF1970). The Science and Technology Bureau of Sichuan province did not have any role in the design of the study and collection, analysis, and interpretation of data and in writing the manuscript.

## Conflict of interest

The authors declare that the research was conducted in the absence of any commercial or financial relationships that could be construed as a potential conflict of interest.

## Publisher's note

All claims expressed in this article are solely those of the authors and do not necessarily represent those of their affiliated organizations, or those of the publisher, the editors and the reviewers. Any product that may be evaluated in this article, or claim that may be made by its manufacturer, is not guaranteed or endorsed by the publisher.
